# Phenetic and genetic structure of tsetse fly populations (*Glossina palpalis palpalis*) in southern Ivory Coast

**DOI:** 10.1186/1756-3305-5-153

**Published:** 2012-07-30

**Authors:** Dramane Kaba, Sophie Ravel, Geneviève Acapovi-Yao, Philippe Solano, Koffi Allou, Henriette Bosson-Vanga, Laetitia Gardes, Eliezer Kouakou N’Goran, Christopher John Schofield, Moussa Koné, Jean-Pierre Dujardin

**Affiliations:** 1 Institut Pierre Richet / Institut National de Santé Publique, BP V 47 Abidjan, Côte d’Ivoire; 2 IRD UMR 177, Laboratoire de Recherche et de Coordination sur les Trypanosomoses IRD-CIRAD, Campus International de Baillarguet, 34398 Montpellier cedex 5, France; 3 Laboratoire de Zoologie, Université d’Abidjan-Cocody, 22 BP 582, Abidjan 22, Côte d’Ivoire; 4 IRD/CIRDES, UMR 177 IRD/CIRAD INTERTRYP, BP 454, 01 Bobo-Dioulasso, Burkina Faso; 5 LSHTM (ITD), London WC1E7HT, UK; 6 IRD, UMR 5090 MIVEGEC, Avenue Agropolis, IRD, Montpellier, France

## Abstract

**Background:**

Sleeping sickness, transmitted by *G. p. palpalis*, is known to be present in the Ivory Coast. *G. p. palpalis* has recently been reported to occur in several places within the town of Abidjan, including: (i) the Banco forest, (ii) the Abobo Adjamé University campus and (iii) the zoological park. Could these three places be treated sequentially, as separate tsetse populations, or should they be taken as one area comprising a single, panmictic population?

**Methods:**

The amount of gene flow between these places provides strategic information for vector control. It was estimated by the use of both microsatellite DNA and morphometric markers. The idea was to assess the interest of the faster and much less expensive morphometric approach in providing relevant information about population structure. Thus, to detect possible lack of insect exchange between these neighbouring areas of Abidjan, we used both genetic (microsatellite DNA) and phenetic (geometric morphometrics) markers on the same specimens.

Using these same markers, we also compared these samples with specimens from a more distant area of south Ivory Coast, the region of Aniassué (186 km north from Abidjan).

**Results:**

Neither genetic nor phenetic markers detected significant differentiation between the three Abidjan *G. p. palpalis* samples. Thus, the null hypothesis of a single panmictic population within the city of Abidjan could not be rejected, suggesting the control strategy should not consider them separately. The markers were also in agreement when comparing *G. p. palpalis* from Abidjan with those of Aniassué, showing significant divergence between the two sites.

**Conclusions:**

Both markers suggested that a successful control of tsetse in Abidjan would require the three Abidjan sites to be considered together, either by deploying control measures simultaneously in all three sites, or by a continuous progression of interventions following for instance the "rolling carpet" principle. To compare the geometry of wing venation of tsetse flies is a cheap and fast technique. Agreement with the microsatellite approach highlights its potential for rapid assessment of population structure.

## Background

Tsetse flies (Diptera: Glossinidae) are the main vectors of trypanosomes (Kinetoplastida: Trypanosomatidae), which cause human and animal trypanosomiases in subsaharan Africa. These diseases have a considerable impact on public health and economic development [1], although there are recent signs of a decline in incidence of the human disease following WHO-supported interventions based on case detection and treatment [2-4]. Vector control is an important complement to case detection and treatment, because reducing vector density can rapidly halt human trypanosomiasis transmission [5,6]. Vector control also remains the only strategy able to protect humans from acquiring a new infection [7].

Tsetse populations may be reduced using a variety of techniques, including insecticide impregnated traps and targets, live-baits, sequential aerial spraying, and sterile male release [8-13]. However, in many cases when the control efforts have been stopped, the tsetse populations tend to recover due to flies surviving the initial interventions, or migrant flies coming from untreated regions, or both.

This has fueled debate as to whether in some instances "eradication" (defined by FAO as the creation of a tsetse free zone) may be more cost-effective than "suppression" where tsetse densities are reduced to a level minimizing the risk of disease transmission. Decisions on eradication or suppression strategies will be facilitated when the population structure within the target region, in particular the degree of genetic isolation of the target population from adjacent populations is clearly understood [14]. For isolated populations, eradication may be the most cost-effective strategy, as reported for *Glossina austeni* Newstead on Unguja Island, Zanzibar [9]. But for most mainland populations of tsetse, the geographical limits of target tsetse populations are less easily defined. Application of techniques that can detect population isolation such as molecular or morphometric markers can guide decisions on the choice of control strategies [15-17]. Human and animal trypanosomiasis transmitted by *G. p. palpalis* are known to be present in Ivory Coast [4,18] and *G. p. palpalis* has been reported to occur within the city of Abidjan [19,20]. Due to its potential danger as a vector of human and animal trypanosomiasis, the Ivorian authorities now seek to control these tsetse flies in the affected area of Abidjan, which includes the Banco forest, the University of Abobo Adjamé and the zoological park. Tsetse have been found to be present in low to high densities in these 3 sites, and were found infected by various trypanosome species [19].

To detect possible evidence of isolation between *G. p. palpalis* populations in the three affected areas within Abidjan, we used both genetic (microsatellite DNA) and phenetic (geometric morphometrics) markers on the same specimens, and compared these populations to *G. p. palpalis* populations from another area of southern Ivory Coast in the region of Aniassué. The idea was to assess the interest of the faster and much less expensive morphometric approach in providing relevant information about population structure.

The expected outcome of this study was to help the national control program to decide which is the best strategy of vector control in the town of Abidjan: can these three localities be treated sequentially (i.e. are the tsetse populations isolated between the three sites), or should they be taken as one area comprising a single, panmictic population?

## Results and discussion

### Microsatellite DNA markers

#### Within sample analyses

For the total sample (n = 141) of genotyped tsetse, the seven microsatellite loci displayed 17 (Pgp1), 17 (PgP13), 14 (PgP24), 25 (B104), 19 (B110), 7 (C102), and 9 (GPCAG) alleles, respectively. The mean number of alleles was 9.71 (Banco), 11 (University) and 10.85 (Zoo) in Abidjan, and 10.00 in Aniassué. Mean observed heterozygosities were 0.68, 0.76 and 0.77 for Banco, University and Zoo, respectively, and 0.70 in Aniassué (no significant difference).

**Figure 1:**
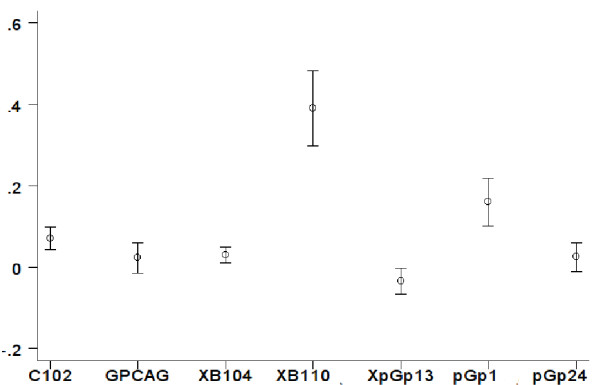
***F***_**is**_**statistics for *Glossina palpalis palpalis *collected from sites of Abidjan.***Fis* statistics for *Glossina palpalis palpalis* collected from sites of Abidjan. For each locus, the *Fis* mean value (circle) is presented with its standard deviation (vertical bar).

Overall *F*_is_ values were 0.12, 0.09 and 0.05 for Banco, University, and Zoo, significant at p<0.0001, p<0.001, and p<0.05 respectively. In Aniassué, *F*_is_ was 0.15, p<0.0001. The heterozygote deficit was mainly due to two loci (PgP1 and B110) for the three populations of Abidjan (Figure 1).

This suggested locus had specific technical problems (e.g. null alleles or short allele dominance), because when these loci were removed from the analysis, *F*_is_ values dropped to non-significant values (0.04, 0.00 and 0.03, respectively). Hence the null hypothesis of panmixia in Abidjan could not be rejected. In Aniassué, *F*_is_ on these 5 loci was 0.18 (p<0.0001), indicating consistant heterozygote deficiency. The heterozygote deficiency found in Aniassué confirmed earlier observations on *G. p. palpalis* in the forested areas of Ivory Coast, which attributed such deficiency to a combination of null alleles and genetic structuring at local scale due to Wahlund effects [21].

#### Genetic differentiation between samples

The mean *F*_st_ value for the 5 loci among the four populations was estimated at *θ* = 0.017 (CI95: 0.011 <*θ*< 0.023), p<0.0001. For the Abidjan samples it was *θ* = 0.007 (CI95: 0.00150 <*θ*< 0.01184) and was not significant, meaning that most of the differentiation was due to differences between Aniassué and Abidjan. Looking at paired *F*_st_ values between sites (Table 1) confirmed this, since the highest (and significant) values always included Aniassué. Within Abidjan, there was a slight but non-significant trend for the population of Banco to diverge (*F*_st_=0.01, p<0.05) from those of University and Zoo, whereas the latter two were genetically similar.

**Table 1:** Metric and genetic distances between sites

**Population 1**	**Population 2**	**Mahalanobis**	***F***_**st**_
Aniassué	Banco	2.38	0.0221
Aniassué	University	1.93	0.0328
Aniassué	Zoo	1.98	0.0292
Banco	University	1.3	0.0121
Banco	Zoo	1.1	0.0113
University	Zoo	0.31	-0.0034

Pairwise metric (Mahalanobis) and genetic (*F*_st_) distances between Banco, University, Zoo (Abidjan) and Aniassué.

### Geometric morphometrics

#### Size: centroid size

**Figure 2:**
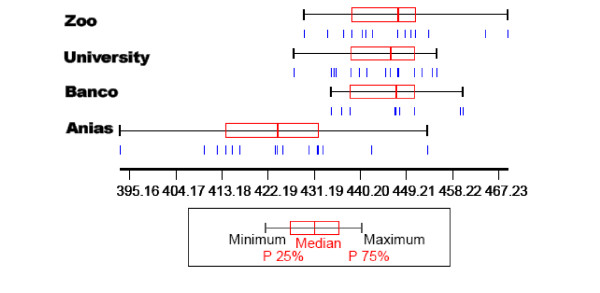
**Size variation of the wings.** Variation of the centroid size of the wing of male *Glossina palpalis palpalis* according to localities. Anias, Aniassué. Each box shows the group median separating the 25th and 75th quartiles. Vertical bars under the boxes represent the wings. Units are pixels. P, percentile.

The specimens from Aniassué were significantly smaller compared to those from Abidjan, whereas within Abidjan there was no significant size difference between flies from the three sites (Figure 2).

#### Shape variation

**Figure 3:**
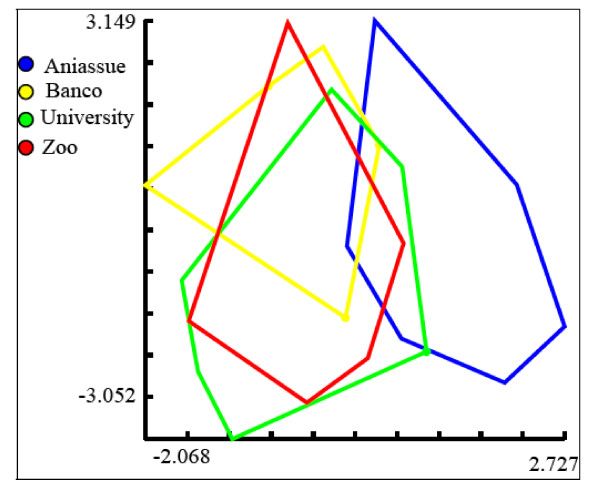
**Morphospace derived from the shape of the wings.** Morphospace of the wings of male *Glossina palpalis palpalis*, the horizontal axis is the first discriminant factor, the vertical axis is the second one. Together, they contributed to of 99% of the total variation.

**Figure 4:**
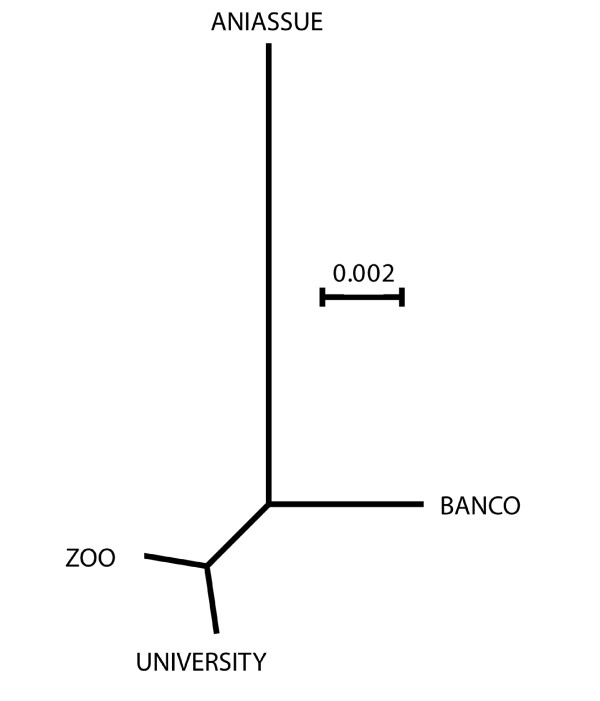
**Classification tree based on the shape of the wings.** Unrooted neighbor joining tree based on the Procrustes distances between *G. p. palpali*s wings from four localities.

The first two discriminant factors derived from the shape variables showed that the polygon representing the Aniassué population tended to separate from the Abidjan sites (Figure 3). The reclassification tree, based on all three of the discriminant factors, clearly separated the Aniassué sample from those from Abidjan (Figure 4).

The Mahalanobis distances between the Abidjan samples (Table 1) were not significantly different, indicating an absence of shape differentiation, while the Mahalanobis distances from Aniassué were significantly larger (p<0.007) (Table 1).

The validated reclassification scores confirmed this pattern, since Aniassué had the highest score (86%). However, in spite of the lack of significant differentiation within Abidjan, the reclassification score obtained for Banco (77%) was much higher than for the University (37%) and Zoo (33%), suggesting a relatively higher level of shape divergence in the Banco forest.

**Figure 5:**
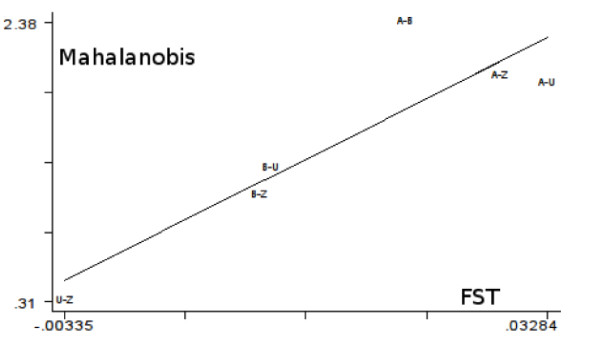
**Correlation between *F***_**st**_**and Mahalanobis distances.** Correlation between *F*_st_ and Mahalanobis distances. Coefficient of determination is 79%. A, Aniassué; B, Banco; U, University; Z, Zoo.

Correlation between metric and genetic distances was high. Regression of the Mahalanobis distances on the genetic distances indicated that 79% of the morphometric variation could be explained by the genetic variation (Figure 5).

### Genetic and morphometric differentiation

From an epidemiological point of view, our study aimed at knowing whether tsetse populations from three sites in Abidjan could be considered to be isolated from each other. Such information is relevant for designing an adequate tsetse control strategy. For example, an insecticide application could be sequential in case of separation between sites, working on each site separately without risk of reinvasion to the next, or it should simultaneously cover all three sites if no evidence for separation is found.

We used a population genetics approach [7] to analyse possible separation between the three Abidjan populations, comparing genetic and phenetic markers. Thus, the study also tested the potential of geometric morphometrics as a possible surrogate for molecular markers.

Both the phenetic (geometric morphometrics) and genetic (microsatellite loci) markers showed no evidence for differentiation between *G. p. palpalis* from sites within Abidjan, but both markers agreed in showing strong differentiation between individuals from Aniassué and those from Abidjan.

#### Within Abidjan

At the scale of Abidjan, our data showed that males from the three sites showed no genetic differentiation, and accordingly had similar metric properties (size and shape).

The microsatellite markers did not show any significant departure from the null hypothesis of panmixia, i.e. we did not observe any genetic differentiation between the 3 populations within Abidjan. There was however a slight, non significant trend for the population of Banco to diverge from the two others. A possible explanation is then a slow, on-going process for this population of Banco to have less genetic exchanges with the two others, due to urbanization which restricts tsetse movements. It may be possible, as observed in other studies in Burkina Faso, that the molecular markers used are not sensitive enough to detect it, since this is a recent, on-going phenomenon whereas what the molecular markers show is the result of a genetic history over several generations. This lack of sensitivity of molecular markers for recent genetic changes has already been observed in tsetse studies [22], and may be compensated by the use of morphometrics.

This idea is reflected by the much higher shape-based reclassification score obtained for Banco (77%), compared to the two other sites (37% and 33%). This indirect evidence for some morphometric specificity in the forest might be due to an environmental effect ("forest" versus "city"), although in tsetse most of the pre-imago development is relatively protected from external influences as tsetse larvae grow in the uterus of their mother during the three first stages, buffering morphometric variations against external influences [23].

Temperature and humidity do become influential factors at the time when pupae are in the soil. The effect has been studied for size (not shape), indicating that higher temperatures tend to result in smaller individuals [23], whereas increasing humidity tends to result in larger individuals [24]. It has been shown that the size of *G. p. palpalis* in forested areas of Ivory Coast is governed by seasonal climatic effects [25]. In Abidjan, no size difference was detected between sites, and given their proximity it seems likely that environmental factors acted uniformly on size.

#### Between Abidjan and Aniassué

By contrast, both molecular (microsatellite loci) and morphometric (centroid size and shape variables) data showed significant differences between tsetse from Abidjan and Aniassué. This was expected due to the geographic distance between the two sites (186 km), to the differences of biotopes, and to the fact that the tsetse belt in South Ivory Coast is discontinuous as a consequence of anthropic pressure on habitats.

The tsetse from Aniassué were smaller than those from Abidjan. This was in agreement with both the slightly higher temperature [23] and dryer conditions [24,26] in Aniassué.

The differences between *G. p. palpalis* from Abidjan and Aniassué also involved shape, which may reflect genetic variations [23,27], especially when shape is allometry-free [28-30]. This was confirmed by differences found using microsatellite DNA markers. The parallel between phenetic and genetic markers applied to natural populations is not uncommon [30]; for *G. p. gambiensis*, a similar parallel was observed in natural populations of different biotopes from West Africa [31], Guinea [15], Burkina Faso [32] and Senegal [17]. Here, 79% of the variance in Mahalanobis distance could be "explained" by genetic variation (compared to 50% in study by [17]) study). This correlation does not imply a causal relationship, and could be attributed to both phenetic and genetic distances being related to geographical distances [33].

The heterozygote deficits found in Aniassué confirmed earlier observations on *G. p. palpalis* in the forested areas of Ivory Coast, which attributed such deficits to a combination of null alleles and genetic structuring at local scale due to Wahlund effects [21].

## Conclusions

**Figure 6:**
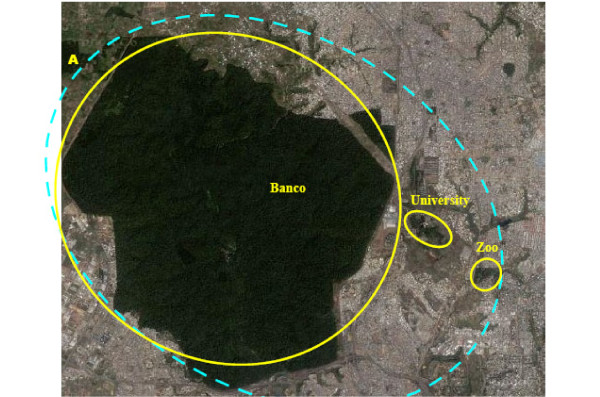
**Eradication strategy.** Eradication strategy by controlling simultaneously the three sites. Blue dotted line: limits of the area to be treated simultaneously. Yellow curves: limits of target sites A: Relic forest of Anguededou not infested by tsetse flies.

**Figure 7:**
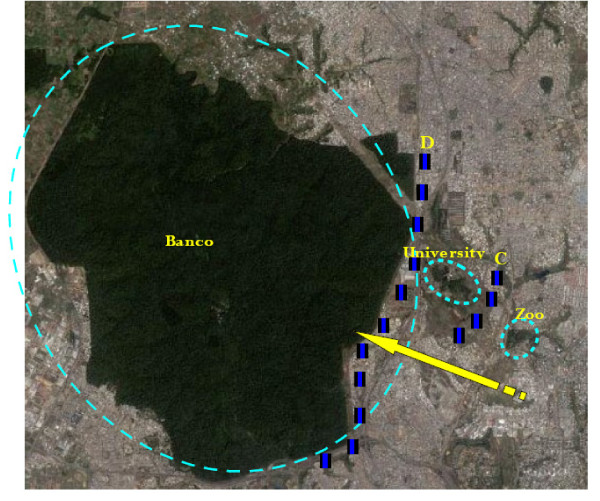
**The "rolling carpet" principle.** Eradication strategy in stages, site after site, but by creating barriers with traps or impregnated screens between Zoo and University (barrier C), University and Banco (barrier B), using the "rolling carpet" principle (Vreysen et al., 2007). The yellow arrow indicates the diection of the steps.

How can the knowledge of population structure help to choose a control strategy? Since microsatellite and morphometric markers did not show significant differentiation between tsetse from the three sites in Abidjan, there would appear to be no significant barrier to gene flow at this scale. From a control perspective, this means that intervention against tsetse in any one site is likely to face reinvasion from the other two. This is different from a similar study conducted on the Loos archipelago, Guinea, which showed that tsetse populations (*G. palpalis gambiensis*) were isolated from the mainland and structured according to the island [15,34], which then allowed a sequential control strategy to be implemented [16,35]. Successful control of tsetse in Abidjan however, would require all three sites to be considered together (Figure 6), either by deploying control measures simultaneously in all three sites, or by a continuous progression of interventions - for example using barriers of impregnated traps and/or targets between sites (Figure 7) following the "rolling carpet" principle [36].

## Methods

### Study area

**Figure 8:**
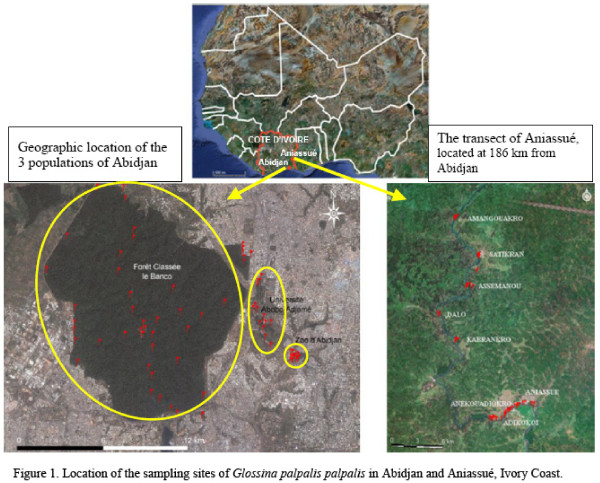
**Geographic area of the study.** Sampling sites of *Glossina palpalis palpalis* in Abidjan and Aniassué, Ivory Coast.

In Abidjan, the three study sites were the Banco forest (Banco), the University of Abobo Adjamé (University) and the zoological park (Zoo). The Banco forest is in the north-western part of the city of Abidjan, at 5°N latitude and 4°W longitude. East of Banco are two small relicts of the forest which have now been substantially degraded by urbanisation: the Abobo Adjamé University and the zoo of Abidjan. These three sites, although geographically close (less than 500 meters between sites), are separated by roads and urbanisation (Figure 8). For comparison, another study site was chosen near the town of Aniassué, about 186 km from Abidjan, in the Department of Abengourou, where *G. p. palpalis* occurs along the Comoé river. This region is characterized by forest degraded by wood cutting, and also by food crops (banana, cassava) and old cocoa plantations.

There is little temperature difference between Abidjan (from 24.2°C to 27.7°C) and Aniassué (from 24.3°C to 27.9°C), but relatively more variation in relative humidity (RH), which decreases from south (RH on average 90%) to north (RH between 60% and 70%). In both areas, there are two rainy and two dry seasons during a year [37].

### Tsetse samples and microsatellite DNA markers

Tsetse flies were caught using Vavoua traps [38] in April 2007 in Aniassué and in October 2007 in Abidjan. In total from Abidjan 111 individual tsetse were analysed using microsatellite DNA markers: Banco (25 females (F), 11 males (M)); University (21 F, 17 M) and Zoo (21 F, 16 M). From Aniassué, 30 individuals were analysed (15 F and 15 M). Seven microsatellite markers were used (preceded by "X" for X-linked loci): Pgp1, XPgp13, Pgp24 [39], XB104, XB110, C102 (A. Robinson, FAO/IAEA, pers. com.) and GPCAG [40]. The samples were processed for Polymerase Chain Reaction (PCR) and genotyping on a 4300 DNA Analysis System from LI-COR (Lincoln, NE) as described in [34].

### Population genetics analyses on molecular markers

Wright's F-statistics [41], the parameters most widely used to describe population genetic structure, were initially defined for a three-level hierarchical population structure (individuals, sub-populations, and total). In such a structure, three fixation indices or F-statistics can be defined.

*F*_is_ is a measure of the inbreeding of individuals (hence I) resulting from non-random union of gametes within each sub-population (hence S).

*F*_st_ quantifies the differentiation between subpopulations in the total population (hence S and T) as a measure of the relatedness between individuals resulting from non-random distribution of individuals between sub-populations, relative to the total population.

*F*_it_ is a measure of the inbreeding of individuals resulting both from non-random union of gametes within sub-populations, and from population structuring (deviation from panmixia of all individuals of the total population, hence I and T).

These F-statistics were estimated by Weir and Cockerham's unbiased estimators *f * (for *F*_is_), *θ* (for *F*_st_) and *F* (for *F*_it_) [42]. The significance of the F-statistics was tested by 1000 random permutations in each case. The significance of *F*_is_ was tested by randomizing alleles between individuals within sub-samples. The significance of *F*_st_ was tested by randomizing individuals among sub-samples.

### Geometric morphometrics analyses

The tsetse specimens used for geometric morphometrics constituted a subsample of those on which the molecular analyses were done. Out of the 141 flies used for microsatellites, 55 had non-damaged wings allowing morphometric analyses. The analyses were conducted only on males, and focused on the right wing, which was generally the wing in best conditions. A total of 55 right wings of *G. p. palpalis* males (M) were used, i.e. 9 from Banco, 16 from University, 15 from Zoo and 15 from Aniassué.

**Figure 9:**
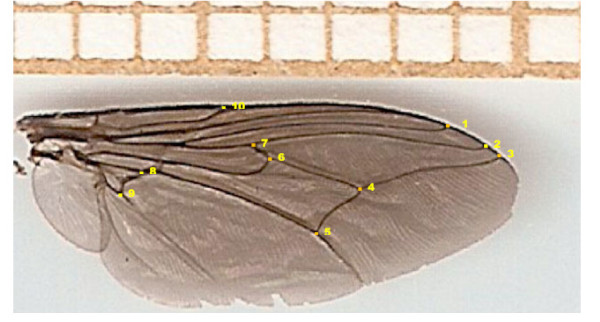
**Anatomical landmarks of the wing.** Ten landmarks at the junction of different veins in the wing of *Glossina palpalis palpalis*. Scale indicates millimeters.

Wings were dry-mounted between two microscope slides and scanned at 1800 ppp at dimensions of 0.90 x 0.50 cm, using a multifunction scanner HP Deskjet F 2180. From this picture, the coordinates of 10 landmarks (LM) defined by vein intersections were recorded for each wing, by the same person in the same order (Figure 9). Repeatability was estimated at better than 80% (discussed elsewhere: [43]).

Raw coordinates were superimposed using the Generalized Procrustes Analysis (GPA) [44,45], producing one variable for size and 16 variables for shape.

The size variable was the isometric estimator known as centroid size (CS) derived from coordinate data and defined as the square root of the sum of the squared distances between the center of the configuration of landmarks, and each individual landmark [46]. Statistical significance for size comparisons was estimated by 1,000 permutation tests [47] with Bonferroni correction.

The 16 shape variables were the "partial warps" (PW). To circumvent the problem of small sample sizes relative to the large number of shape variables (16 PW), we used the first 6 principal components of the PW (relative warps, RW) as input for discriminant analyses, as these represented 84% of the total variation and had the highest discriminatory power [48].

Mahalanobis distances [49] computed from these 6 RW were used to quantify shape divergence between groups (Figure 4) and the statistical significance was estimated by 1000 permutation tests [50] with Bonferroni correction.

Mahalanobis distances based re-classification scores were computed according to a validation procedure whereby each individual was assigned to its closest group without using that individual to help determine a group centre [33], although the computed shape variables did include that individual [43] (Table 2).

**Table 2:** Reclassification of tsetse individuals based on the shape of the wings

**Populations**	**Correctly assigned individuals**	
Aniassué	13 / 15	86%
Banco	7 / 9	77%
University	6 / 16	37%
Zoo	5 / 15	37%

Validated reclassification of tsetse individuals based on the shape of their wings.

### Software

Collections of anatomical landmarks of the wings, general Procrustes analysis (GPA), multivariate and discriminant analyses, were performed using the CLIC package [43], freely available at http://www.mpl.ird.fr/morphometrics/clic/index.html. PHYLIP software with "neighbor" module [51] and NJPLOT [52] were used to build the classification tree. The F-statistics from molecular data were estimated with Genetix [53] and Fstat 2.9.3.2 (updated from [54]). The overall G-test was used to estimate the significance of *F*_st_ with Fstat [55].

## Competing interests

The authors declare that they have no competing interests.

## Authors' contributions

Genetic techniques: SR, KA, LG, EKN'G, PS. Morphometric techniques: DK, HB-V, J-PD. Data analyses: DK, SR, PS, J-PD. Field collections: DK, MK, GA-Y. Text: DK, PS, J-PD, CJS. All authors read and approuved the final version of the manuscript.
